# Comparative analysis of frequentist, Bayesian, and machine learning models for predicting SARS-CoV-2 PCR positivity

**DOI:** 10.3389/frai.2025.1668477

**Published:** 2025-12-03

**Authors:** Francis Chukwuebuka Ihenetu, Chinyere Ihuarulam Okoro, Makuochukwu Maryann Ozoude, Emeka H. Okechukwu, Easter Godwin Nwokah

**Affiliations:** 1 Deapartment of Microbiology, Imo State University, Owerri, Imo, Nigeria; 2 Department of Microbiology, Federal Teaching Hospital Owerri, Owerri, Imo, Nigeria; 3 Zaporizhzhia State Medical and Pharmaceutical University, Zaporizhzhia, Ukraine; 4 Department of Medical Laboratory Science, Imo State University, Owerri, Imo, Nigeria; 5 Department of Medical Microbiology, Rivers State University, Port Harcourt, Nigeria

**Keywords:** SARS-CoV-2, PCR testing, logistic regression, Bayesian analysis, random forest, predictive modeling

## Abstract

**Background:**

Prediction of infection status is critical for effective disease management and timely intervention. Traditional diagnostic methods for Severe Acute Respiratory Syndrome Coronavirus 2 (SARS-CoV-2) are challenged by varying sensitivities and specificities, necessitating the evaluation of advanced statistical approaches. This study evaluated the predictive performance of frequentist logistic regression, Bayesian logistic regression, and a random forest classifier using clinical and demographic predictors to predict PCR positivity.

**Methodology:**

A total of 950 participants were analyzed using three modeling approaches. To address class imbalance, the data were balanced using the Synthetic Minority Oversampling Technique (SMOTE) before training the random forest classifier. Predictors include IgG serostatus, travel history (international and domestic), self-reported symptoms (such as loss of smell, fatigue, sore throat), sex, and age. Three models were developed: (1) frequentist logistic regression; (2) Bayesian logistic regression with a moderately informative Normal (mean = 1, SD = 2) prior and a weakly informative Cauchy (0, 2.5) prior; and (3) machine learning (ML) using a random forest classifier. Missing data were minimal (<2%) and handled through imputation, with sensitivity analyses confirming no material impact on model performance. Performance was evaluated using odds ratios, posterior means with credible intervals, and area under the ROC curve (AUC).

**Results:**

Of the 950 participants, 74.8% tested positive for SARS-CoV-2. The frequentist logistic regression identified recent international travel (Odds Ratio = 4.8), loss of smell (OR = 2.3), and domestic travel (OR = 1.5) as the strongest predictors of PCR positivity. The Bayesian model yielded similar posterior estimates, confirming the robustness of these associations across prior assumptions. The random forest classifier achieved the highest discriminative performance (AUC = 0.947–0.963). Notably, age and sex were not significant in the regression models but emerged as influential predictors in the random forest model, suggesting possible nonlinear or interaction effects.

**Conclusion:**

The machine learning approach (random forest) outperformed the logistic regression models in predictive accuracy. Bayesian regression confirmed the reliability of key predictors and allowed quantification of uncertainty. These findings highlight that simple, routinely collected symptom and exposure data can support rapid, resource-conscious screening for SARS-CoV-2, particularly when laboratory testing capacity is limited.

## 1 Introduction

The unprecedented global pandemic precipitated by SARS-CoV-2 has highlighted the essential role of predictive modeling in public health, particularly in informing interventions and resource allocation. Accurate prediction of PCR test positivity for SARS-CoV-2 is critical as it enables authorities to implement timely measures aimed at curbing virus transmission and managing healthcare resources effectively. Among numerous statistical methodologies employed for binary outcome prediction, logistic regression stands out as a conventional yet powerful tool. Within this framework, both frequentist and Bayesian approaches have been widely utilized, each offering distinct perspectives on data analysis (Hosmer et al., 2013; Gelman et al., 2013; Ihenetu et al., 2024).

Coronavirus disease 2019 (COVID-19) diagnosis relies on nucleic acid amplification tests, particularly RT-PCR testing for SARS-CoV-2, which is widely regarded as the clinical reference standard. However, RT-PCR has practical limitations; it requires laboratory infrastructure, and results often take hours or days to return (Zoabi et al., 2021; Kucirka et al., 2020; Crozier et al., 2021; Wertenauer et al., 2023). In resource-limited settings or peak surges, PCR tests may be scarce or backlogged, delaying identification of infectious individuals. Furthermore, RT-PCR sensitivity varies significantly depending on the stage of infection. Sensitivity is lower during the pre-symptomatic phase, and false-negative results can occur due to suboptimal sampling technique or timing (Zoabi et al., 2021; Kucirka et al., 2020; Jindal et al., 2021; Kanji et al., 2021). These temporal and procedural limitations introduce potential misclassification bias when using PCR as the outcome variable in predictive modeling studies (Kucirka et al., 2020).

Given these constraints, our study does not assume PCR to be a flawless gold standard, but instead uses it as the best available diagnostic benchmark during the time of data collection, integrating clinical and laboratory findings when appropriate. The aim of our study is to augment rather than replace PCR testing by developing a predictive model that can rapidly flag likely positive cases. Such a tool could be used for preliminary screening and triage while awaiting confirmatory PCR, especially when immediate PCR testing is unavailable. We recognize PCR’s limitations and aim to create a model that complements its use by offering rapid, preliminary risk estimation based on clinical data.

Prior research during the pandemic has explored symptom-based screening and risk scores to prioritize testing when capacity is limited (Zhang et al., 2021; Callahan et al., 2020; Lan et al., 2020). These approaches leverage readily available clinical predictors to identify high-risk patients, helping to bridge the gap in settings of limited access or delayed PCR results (Callahan et al., 2020; Wikramaratna et al., 2020; Vandenberg et al., 2020; Baik et al., 2022).

Several studies have reported that combinations of symptoms, exposures, and basic demographics can predict PCR positivity with reasonable accuracy (Elliott et al., 2021; Menni et al., 2020; Elimian et al., 2021; Aung et al., 2024). For example, models asking about key symptoms and risk factors (such as recent exposures or travel) have shown area-under-curve values around 0.8–0.9 for discriminating positive vs. negative cases (Aung et al., 2024; Quer et al., 2020). This suggests that early clinical information can be harnessed to assist PCR testing by indicating which patients are most likely to be infected.

Building on this concept, our study compares three modeling approaches for predicting SARS-CoV-2 PCR results: a traditional frequentist logistic regression, a Bayesian logistic regression, and a machine learning model (random forest). By comparing these approaches, we evaluate whether advanced methods (Bayesian inference or non-linear machine learning) offer any gains in predictive performance or practical insights over the standard logistic model. We specifically focus on a cohort of unvaccinated individuals undergoing PCR testing, using predictors such as self-reported symptoms and recent travel history as predictors. While this enhances internal validity, it restricts external applicability to vaccinated or previously infected populations; a limitation we explicitly address.

The goal is to determine if an interpretable predictive model could serve as an early warning tool to flag likely positive cases for isolation or expedited confirmatory testing, thereby augmenting PCR-based diagnosis in scenarios of limited testing availability or slow turnaround times.

## 2 Materials and methods

### 2.1 Study design

This cross-sectional study was conducted to compare the performance of frequentist, Bayesian, and ML logistic regression models in predicting SARS-CoV-2 PCR positivity. Data were collected from 950 participants at the Federal University Teaching Hospital in Owerri, Imo State, Nigeria, between December 2020 and October 2024, encompassing the peak period of the COVID-19 pandemic. The structured questionnaire used for data collection is available as a Supplementary material S1.

### 2.2 Participants criteria

Individuals presenting for SARS-CoV-2 testing were recruited. Inclusion criteria were: age ≥18 years and presence of symptoms consistent with COVID-19. Individuals with a confirmed prior COVID-19 diagnosis or those who received a COVID-19 vaccination within 2 months prior to enrollment were excluded to minimize biases arising from pre-existing immunity or vaccine-induced serological responses (Yilmaz & Çelik, 2021). This exclusion criterion focused the analysis on a cohort of *de novo* infections, avoiding confounding due to immunity status. All participants provided written informed consent.

### 2.3 Data collection

Trained healthcare personnel administered a structured questionnaire capturing demographic information (age, sex, and marital status), clinical symptoms (fever, cough, sore throat, anosmia, and gastrointestinal symptoms), recent travel history, and exposure to confirmed SARS-CoV-2 cases. Recent travel history was assessed by asking: “Have you traveled outside the country or to a high-risk region in the past 30 days?” Additionally, data on pre-existing comorbidities, including respiratory diseases, were obtained to adjust for confounding variables in the modeling process.

### 2.4 Laboratory testing

Nasopharyngeal swabs were obtained from all participants for SARS-CoV-2 detection using real-time reverse transcription-polymerase chain reaction (rRT-PCR), which is regarded as the gold standard for COVID-19 diagnostics (Dybowski, 2020). In addition, immunological assays were employed to detect IgG antibodies against SARS-CoV-2.

### 2.5 Data preprocessing

Before analysis, the dataset underwent rigorous preprocessing to ensure data quality and integrity. In practice, missing data were minimal (each variable <2%), and were imputed using median values for continuous variables and the mode for categorical variables (Rohmah et al., 2023). To ensure the imputation did not bias results, we conducted a sensitivity analysis comparing model outcomes with and without the imputed data; the key findings remained unchanged, indicating that our imputation approach did not significantly affect the results. Continuous variables were standardized to enhance model convergence and interpretability; in fact, age was the only continuous predictor and it was standardized (*z*-score transformed). Outlier detection was performed using statistical methods (e.g., *Z*-scores, interquartile range) and visual inspection (e.g., boxplots), with corrections made to avoid model distortion. No extreme outliers necessitating removal were found; no data points were excluded, and any mildly outlying values were examined and left as-is or winsorized if needed, as they did not unduly influence the model as histograms of continuous variables (e.g., age) confirmed these were approximately normally distributed. To address the marked class imbalance (74.8% PCR-positive vs. 25.2% PCR-negative), we employed SMOTE using the “DMwR” package in R. This procedure oversampled the minority (PCR-negative) class to achieve a 1:1 ratio prior to model training. Without this balancing step, preliminary models overwhelmingly predicted the majority class (PCR-positive) for most cases, resulting in near-zero sensitivity for PCR-negative cases. Applying SMOTE improved the classifier’s ability to detect the minority class, as evidenced by substantially higher recall and balanced accuracy in the results. Model evaluation emphasized balanced accuracy, precision, recall, and F1-score over raw accuracy to reflect true performance.

*Imputation sensitivity*: To assess the impact of imputation, we repeated all model fits using complete-case data (listwise deletion) and compared performance metrics to those from the imputed dataset. Results were highly consistent (AUC Δ ≤ 0.008; Sensitivity Δ ≤ 0.012; Balanced Accuracy Δ ≤ 0.013), and model ranking was unchanged (Supplementary Table S3), indicating that imputation did not materially affect predictive findings.

### 2.6 Statistical analysis

#### 2.6.1 Variable selection

Variables were selected based on clinical relevance, statistical significance, and insights from existing literature to ensure the inclusion of predictors supported by empirical evidence and theoretical justification (Yilmaz & Çelik, 2021).

#### 2.6.2 Frequentist logistic regression

A frequentist logistic regression model was employed to evaluate the association between SARS-CoV-2 PCR positivity and various clinical and demographic predictors. This model estimates the likelihood of a positive test result based on observed variables, derived from data without integrating prior distributions. Predictor variables were selected based on their clinical significance, statistical significance in univariate analysis (*p*-value < 0.25), and previous findings in the literature (De Smedt et al., 2023; Lukman et al., 2021). A backward stepwise elimination technique refined the model, guided by the Akaike Information Criterion (AIC) for optimal fit. Multicollinearity was assessed using Variance Inflation Factors (VIF), with variables exhibiting high VIF values reviewed and managed to mitigate redundancy (Ihenetu et al., 2024; Tschoellitsch et al., 2020). Outliers and influential observations were identified by examining standardized residuals, leverage statistics, and Cook’s distance, with necessary adjustments made to minimize their influence. The performance of the frequentist model was evaluated using the area under the ROC to gauge predictive accuracy, supplemented by classification tables, sensitivity, specificity, and goodness-of-fit tests for comprehensive model assessment (Couronné et al., 2018). Predictors that fit well into the classic regression were included into the Bayesian model. Additionally, we evaluated a potential non-linear effect of age by including a quadratic age term in preliminary models; this term was not significant (*p* > 0.3) and did not improve model fit, so age was retained as a linear predictor.

#### 2.6.3 Bayesian logistic regression

Bayesian logistic regression was employed to estimate the probability of SARS-CoV-2 PCR positivity while incorporating prior distributions for the regression coefficients. Two models were specified to evaluate the influence of different prior assumptions. In the primary model (Model 1), a moderately informative prior was defined using a normal distribution with mean = 1 and standard deviation = 2, based on previous epidemiological applications (Ihenetu et al., 2024; Lee, 2025; Gelman et al., 2008; Vehtari, 2025; Fleitas et al., 2021). This configuration provided slight regularization and a mild positive bias, allowing prior information to influence estimates without dominating the observed data. To assess the robustness of inferences, a prior sensitivity analysis (Model 2) was conducted using a weakly informative Cauchy prior centered at zero with a scale of 2.5, as recommended by Gelman et al. (2008). This prior is commonly used in Bayesian logistic regression due to its flexibility and ability to down-weight extreme coefficient values. Both models were implemented using the “brms” package in R, leveraging Hamiltonian Monte Carlo sampling. Convergence was assessed using trace plots and the Gelman-Rubin statistic (Rˆ), following standard Bayesian diagnostic criteria. Posterior estimates were summarized using posterior medians and 95% credible intervals (CrI).

#### 2.6.4 Machine learning-based prediction and model interpretation

##### 2.6.4.1 Data preprocessing and class balancing

The dataset was preprocessed to ensure consistent variable encoding. All categorical predictors were converted to factors, and one-hot encoding was applied where appropriate. Before balancing, the model exhibited near-zero sensitivity for PCR-negative cases due to the 3:1 class imbalance; this was corrected after SMOTE (Table 1; Supplementary Table S3). To address class imbalance (711 PCR-positive vs. 239 PCR-negative cases), SMOTE was applied using the “themis” package in R (Table 1) (Ramentol et al., 2011). (Before SMOTE, the dataset contained 711 PCR-positive and 239 PCR-negative cases. After balancing, both classes had 711 observations, ensuring equal representation and reducing bias in model training). Initial model training on the imbalanced dataset (74.8% PCR-positive vs. 25.2% PCR-negative) resulted in very low sensitivity for the negative class, often approaching zero. This occurred because the classifiers, particularly the random forest, optimized overall accuracy by favoring the dominant class. In practice, the models learned to classify most observations as positive, minimizing false negatives at the expense of missing many true negatives. This imbalance-driven bias justified applying SMOTE to synthetically increase minority-class examples and rebalances the training data prior to model fitting. Because SMOTE addresses class imbalance during model training, it was applied only to the Random Forest classifier. Logistic and Bayesian regression were evaluated under the original class distribution (no SMOTE).

**Table 1:** Comparative performance of random forest models with and without travel history.

Metric	With travel (mean ± SE)	Without travel (mean ± SE)
Accuracy	0.786 ± 0.0129	0.812 ± 0.0064
Balanced accuracy	0.648 ± 0.0185	0.813 ± 0.0061
F1 score	0.463 ± 0.0356	0.790 ± 0.0071
Recall (sensitivity)	0.368 ± 0.0321	0.708 ± 0.0156
Specificity	0.927 ± 0.0090	0.917 ± 0.0168
ROC AUC	0.963	0.947
True positives	262	504
False positives	17	20
True negatives	222	219
False negatives	449	207

Metrics were estimated using 5-fold cross-validation on SMOTE-balanced data. Confusion matrix values are totals across folds out of 711 actual positives and 239 actual negatives.

##### 2.6.4.2 Model development and cross-validation

A random forest classifier was developed using the “ranger” engine within the “*tidymodels*” framework in R. Model training and evaluation were based on 5-fold cross-validation applied to the SMOTE-adjusted dataset. Additionally, 20% of the data were set aside as a hold-out test set to validate the final model’s performance on unseen cases.

Model performance was assessed using standard classification metrics derived from the confusion matrix, including accuracy, balanced accuracy, sensitivity (recall), specificity, F1 score, and the area under the ROC curve (AUC).

Performance metrics were calculated as follows:


$$\text{Sensitivity}\;(\text{Recall})=\frac{\mathrm{TP}}{\left(\mathrm{TP}+\mathrm{FN}\right)},\text{Specificity}=\frac{\mathrm{TN}}{\left(\mathrm{TN}+\mathrm{FP}\right)}$$

$$\begin{array}{l} \text{Accuracy}=\frac{\left(\mathrm{TP}+\mathrm{TN}\right)}{\left(\mathrm{TP}+\mathrm{TN}+\mathrm{FP}+\mathrm{FN}\right)},\;\\ \text{Balanced Accuracy}=\frac{\left(\text{Sensitivity}+\text{Specificity}\right)}{2} \end{array}$$

$$\text{Precision}=\frac{\mathrm{TP}}{\left(\mathrm{TP}+\mathrm{FP}\right)},F1=\frac{2\times (\text{Precision}\times \text{Recall})\;}{\left(\text{Precision}+\text{Recall}\right)}$$

ROC AUC values were obtained using the average of the sensitivity-specificity trade-offs across thresholds. The confusion matrix terms are defined as:

- TP (True Positives) = correctly identified PCR-positive cases.
- TN (True Negatives) = correctly identified PCR-negative cases.
- FP (False Positives) = PCR-negative cases incorrectly predicted as positive.
- FN (False Negatives) = PCR-positive cases incorrectly predicted as negative.

All performance metrics were computed using the test set predictions for both Random Forest models (with and without travel history), and confusion matrix counts (TP, FP, TN, FN) were used to verify metric consistency. To assess model robustness, a second random forest model was trained after removing international and domestic travel history from the predictor set, allowing direct comparison of predictive performance with and without travel variables (Ramentol et al., 2011).

##### 2.6.4.3 Model explainability using SHAP values

To enhance model interpretability, SHAP (SHapley Additive exPlanations) values were computed using the “*iml*” package. SHAP summary plots were generated to visualize the relative contribution of each predictor to the random forest model’s output. Two SHAP analyses were performed; one using all predictors, and another excluding travel-related variables; to highlight differences in predictor importance across models (Kivrak et al., 2024).

##### 2.6.4.4 Model comparison and sensitivity analysis

Comparative evaluation of the random forest models; with and without travel-related predictors; was performed using 5-fold cross-validated metrics, confusion matrices, and ROC curves. This analysis assessed the effect of excluding travel history (international and domestic) on model performance. The models were trained on the same SMOTE-adjusted dataset to ensure fair comparison of predictive power between full and reduced variable sets.

### 2.7 Software and computational tools

All statistical analyses were conducted using RStudio (v4.5.1) and JASP (v0.19.3). Bayesian logistic regression models were implemented entirely in R using the “*brms*” package. Classical logistic regression was performed in JASP and independently confirmed in R for consistency. Machine learning procedures, including SMOTE balancing and Random Forest modeling, were executed using the “*tidymodels*,” “*ranger*,” “*themis*,” and “*iml*” packages in R. All figures were generated in JASP or R, as appropriate.

### 2.8 Ethical considerations

Ethical approval was obtained from the Institutional Review Board of the Federal University Teaching Hospital, Owerri. Informed consent was secured from all participants prior to data collection and PCR testing. All data were anonymized, and confidentiality was maintained throughout the study in compliance with ethical research standards (De Smedt et al., 2023).

## 3 Results

Table 2 presents the descriptive characteristics of the study population stratified by PCR status. The mean age was similar between PCR-positive (36.8 years) and PCR-negative (38.2 years) participants. Females represented approximately two-thirds of both groups. Most participants were married, but widowed individuals were more common among PCR-negative cases (6.3%) compared to positives (2.3%). Symptom profiles differed between groups. Fatigue was more common among PCR-negative participants (90.8%) than PCR-positive (84.4%), while loss of smell showed a stronger association with positivity—reported in 85.5% of PCR-positive individuals versus 71.5% of PCR-negatives. Other symptoms like abdominal pain, cough, and runny nose were frequent in both groups but slightly more prevalent in PCR-positive cases. Travel history was notably different. A larger proportion of PCR-positive individuals had recent international travel (92.4%) compared to PCR-negative (71.5%). A similar trend was seen for domestic travel (69.1% vs. 59.4%). IgG seropositivity was more frequent in PCR-positive participants (26.4%) than PCR-negative (20.1%). History of respiratory disease was rare and similar across both groups.

**Table 2:** Descriptive characteristics of the study population by PCR test result. (PCR-positive vs. PCR-negative groups).

Covariate	Category	PCR positive (*N* = 711) (%)	PCR negative (*N* = 239) (%)
Age	Mean (± SD)	36.8 ± 15.3	38.2 ± 15.7
PCR test result	Positive (%)	711 (100%)	—
	Negative (%)	—	239 (100%)
IgG serostatus	Positive	188 (26.4%)	48 (20.1%)
	Negative	523 (73.6%)	191 (79.9%)
Sex	Female	466 (65.5%)	157 (65.7%)
	Male	245 (34.5%)	82 (34.3%)
Marital status	Married	440 (61.9%)	161 (67.4%)
	Single	255 (35.9%)	63 (26.4%)
	Widow/widower	16 (2.3%)	15 (6.3%)
Fatigue	Yes	600 (84.4%)	217 (90.8%)
	No	111 (15.6%)	22 (9.2%)
Loss of smell	Yes	608 (85.5%)	171 (71.5%)
	No	103 (14.5%)	68 (28.5%)
Runny nose	Yes	573 (80.6%)	178 (74.5%)
	No	138 (19.4%)	61 (25.5%)
Shortness of breath	Yes	586 (82.4%)	197 (82.4%)
	No	125 (17.6%)	42 (17.6%)
Abdominal pain	Yes	535 (75.2%)	155 (64.9%)
	No	176 (24.8%)	84 (35.1%)
Respiratory disease history	Yes	28 (3.9%)	10 (4.2%)
	No	683 (96.1%)	229 (95.8%)
Cough	Yes	522 (73.4%)	181 (75.7%)
	No	189 (26.6%)	58 (24.3%)
Sore throat	Yes	570 (80.2%)	188 (78.7%)
	No	141 (19.8%)	51 (21.3%)
Domestic travel (30 days)	Yes	491 (69.1%)	142 (59.4%)
	No	220 (30.9%)	97 (40.6%)
International travel (30 days)	Yes	657 (92.4%)	171 (71.5%)
	No	54 (7.6%)	68 (28.5%)

All percentages are calculated within each PCR outcome group.

A supplementary file has been added to summarize the rate of missingness for each variable. All variables had less than 2% missing data. Imputation methods and summary counts are explicitly presented in Supplementary Table S2.

The logistic regression model including the selected predictors (Model 1) significantly improved the fit compared to the null model (Δ*χ*^2^ = 164.352, *p* < 0.001), as shown in Table 3. The model explained a modest proportion of the variance in PCR test positivity (McFadden *R*^2^ = 0.153; Nagelkerke *R*^2^ = 0.235), consistent with expectations for clinical prediction models.

**Table 3:** Frequentist logistic regression model summary for predicting SARS-CoV-2 PCR positivity.

Model	Deviance	AIC	BIC	df	Δ*χ*^2^	*p*	McFadden *R*^2^	Nagelkerke *R*^2^	Tjur *R*^2^	Cox & snell *R*^2^
M₀	1071.720	1073.720	1078.576	949	—	—	—	—	—	—
M₁	907.368	939.368	1017.071	934	164.352	<0.001	0.153	0.235	0.191	0.159

Table 4 shows the final stepwise logistic regression model estimates. Several variables emerged as significant predictors of PCR positivity. Reporting fatigue was associated with decreased odds of a positive PCR result (coef = −0.617, *p* = 0.021, 95% CI: −1.142 to −0.092). Conversely, reporting loss of smell was associated with higher odds of testing positive (coef = 0.849, *p* = 0.026, 95% CI: 0.099 to 1.599). Participants with abdominal pain were also more likely to test positive (coef = 0.486, *p* = 0.030, 95% CI: 0.048 to 0.924). Domestic travel in the past 30 days was a significant predictor (coef = 0.419, *p* = 0.025, 95% CI: 0.054 to 0.785). Notably, recent international travel was the strongest predictor in the model (coef = 1.566, *p* < 0.001, 95% CI: 1.080 to 2.052).

**Table 4:** Stepwise logistic regression model for predicting SARS-CoV-2 PCR positivity in the model selection process.

Predictor	Estimate	SE	Wald *χ*^2^	df	*p*	95% CI	
						Lower	Upper
M₀ (Intercept)	1.090	0.075	212.600	1	<0.001	0.944	1.237
M₁ (Intercept)	−1.221	0.449	7.378	1	0.007	−2.101	−0.340
Fatigue (Yes)	−0.617	0.268	5.298	1	0.021	−1.142	−0.092
Loss of smell (Yes)	0.849	0.383	4.924	1	0.026	0.099	1.599
Abdominal pain (Yes)	0.486	0.223	4.733	1	0.030	0.048	0.924
Domestic travel (Yes)	0.419	0.186	5.057	1	0.025	0.054	0.785
International travel (Yes)	1.566	0.248	39.845	1	<0.001	1.080	2.052

Table shows variables retained in the final stepwise logistic regression model. Other predictors were excluded during the selection process.

**Figure 1:**
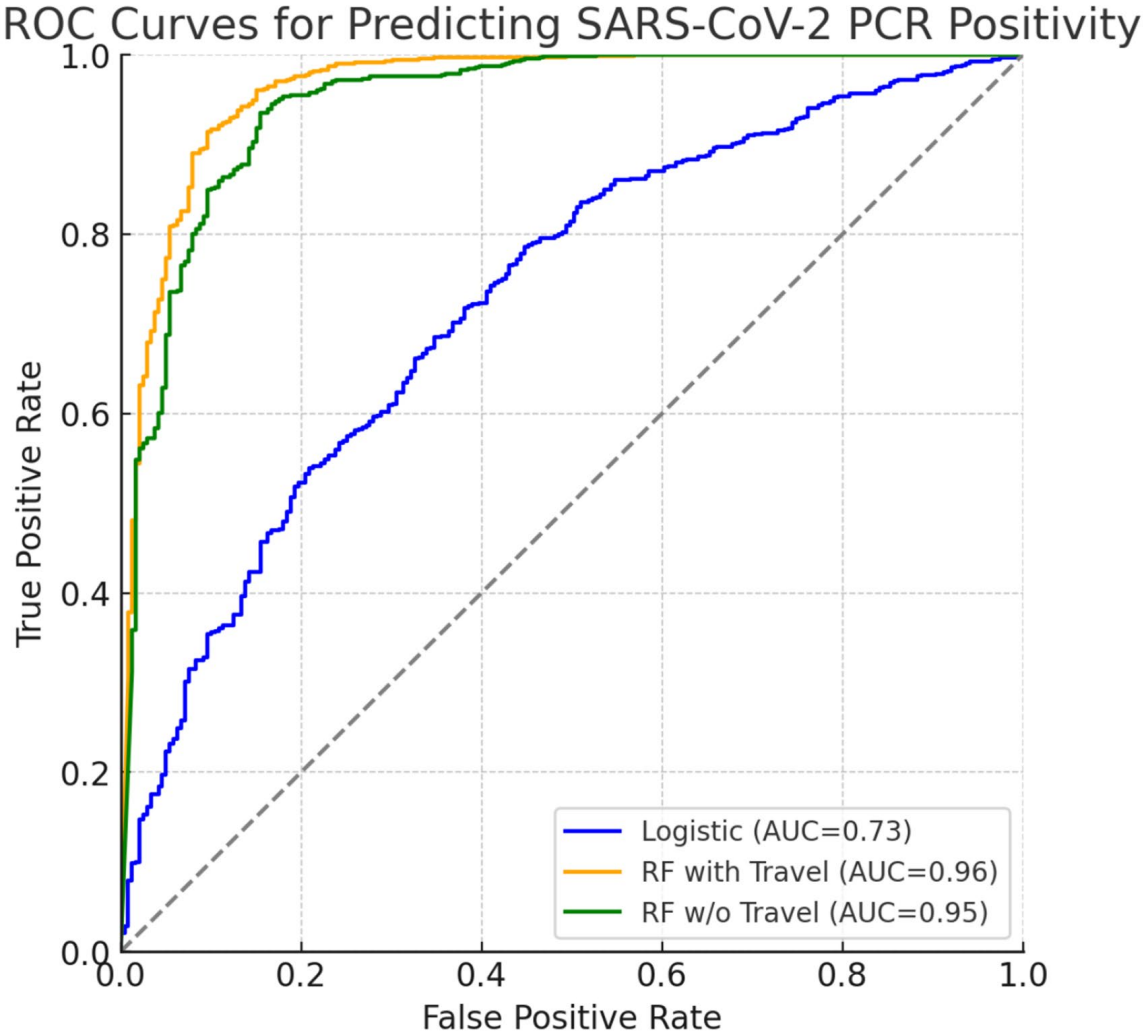
ROC curves comparing predictive models for SARS-CoV-2 PCR positivity. The blue curve represents the frequentist logistic regression model (AUC = 0.73), the orange curve represents the random forest model including travel history (AUC = 0.963), and the green curve represents the random forest model excluding travel history (AUC = 0.947). Both axes range from 0.0 to 1.0.

The frequentist logistic regression model achieved an overall accuracy of 0.797 and an AUC of 0.728, reflecting moderate discriminative ability. Sensitivity was high (0.961), but specificity was lower (0.310), indicating strong case detection but limited ability to rule out negatives. Detailed performance metrics are shown in Table 5, and the AUC is visualized in Figure 1 alongside the Bayesian and random forest models.

**Table 5:** Performance diagnostics of the frequentist logistic regression model.

Performance metric	Value
Accuracy	0.797
AUC	0.728
Sensitivity	0.961
Specificity	0.310
Precision	0.805

**Figure 2:**
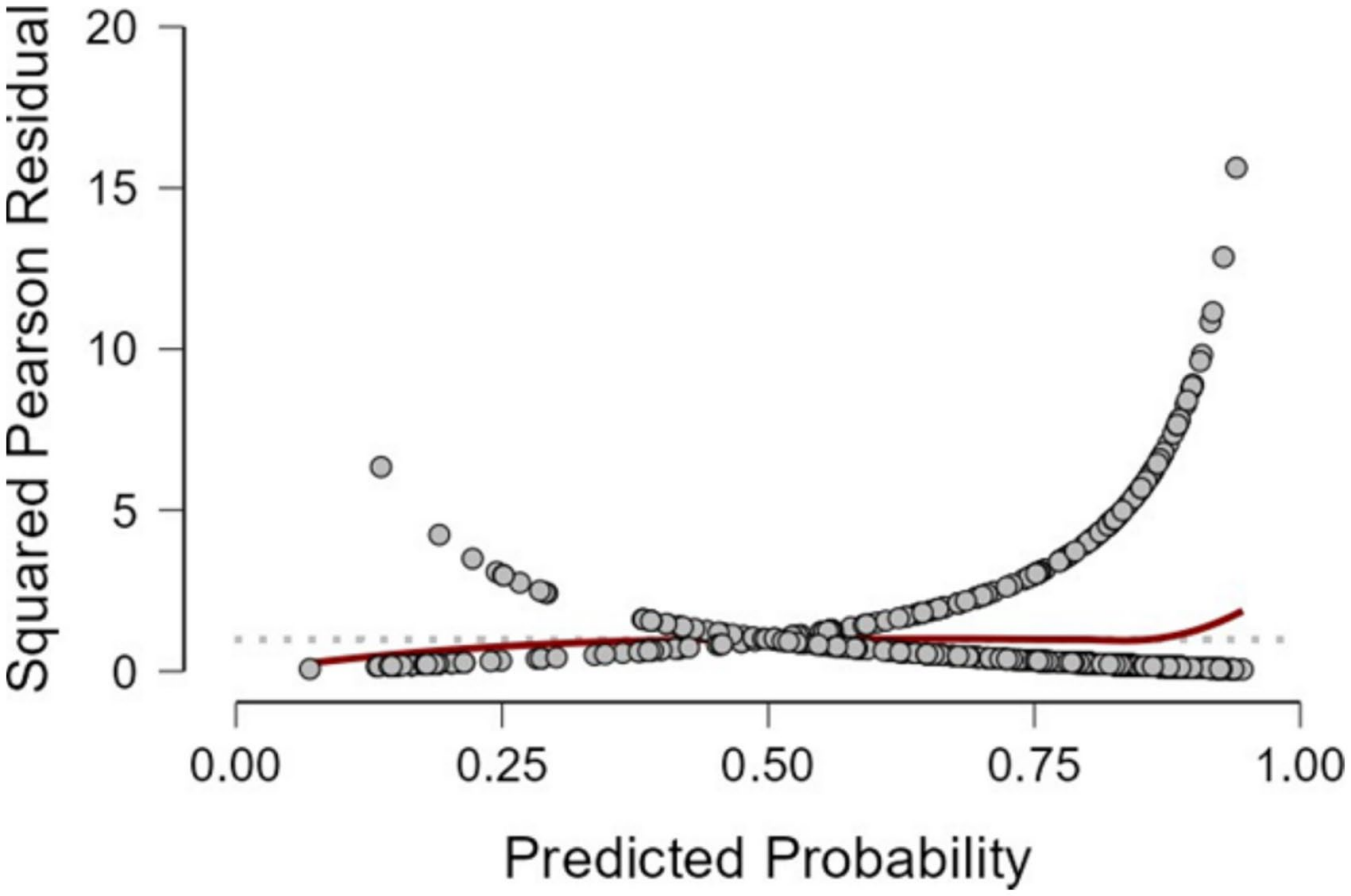
Squared Pearson residuals plot for the frequentist logistic regression model.

Figure 2 displays the distribution of squared Pearson residuals from the frequentist logistic regression model. Most residuals are concentrated near the lower end of the scale, with no discernible outliers or influential data points. The smooth trend line remains close to zero across predicted probabilities, indicating no major departures from model assumptions and an adequate overall fit.

The Bayesian analysis largely corroborated the frequentist results. Table 6 summarizes the posterior estimates from Model 1 (with moderately informative Normal priors). Consistent with the classical model, loss of smell (posterior mean = 0.85; 95% CrI: 0.08 to 1.61), abdominal pain (0.49; 95% CrI: 0.04 to 0.91), domestic travel (0.42; 95% CrI: 0.10 to 0.77), and international travel (1.61; 95% CrI: 1.14 to 2.07) were significant positive predictors of SARS-CoV-2 positivity. Fatigue was negatively associated with PCR positivity (−0.59; 95% CrI: −1.15 to −0.07). Predictors such as sex, age, sore throat, cough, and history of respiratory disease had 95% credible intervals that included zero, suggesting weak or uncertain associations. These Bayesian credible intervals (CrIs) reinforce which effects are credibly non-zero and mirror the confidence interval findings of the frequentist model. The use of a weakly informative Cauchy prior (Model 2) produced very similar results (e.g., international travel posterior = 1.60, 95% CrI: 1.11 to 2.10; loss of smell = 0.85, 95% CrI: 0.08 to 1.58; abdominal pain = 0.48, 95% CrI: 0.03 to 0.92; domestic travel = 0.42, 95% CrI: 0.09 to 0.77; fatigue = −0.61, 95% CrI: −1.13 to −0.09), indicating that our choice of prior did not materially affect the findings. All Markov chain diagnostics were satisfactory (Rˆ = 1.00).

**Table 6:** Posterior summaries from Bayesian logistic regression model 1 (moderately informative priors).

	Coefficient	Mean	SD	95% CI Lower	95% CI Upper
1	(Intercept)	−1.2977	0.4608	−2.2171	−0.3970
2	Age	0.0015	0.0067	−0.0117	0.0144
3	Sex (male)	0.2342	0.1827	−0.1219	0.5989
4	Marital_status1 (single)	0.3129	0.2162	−0.1063	0.7441
5	Marital_status2 (widow/widower)*	−0.8371	0.4197	−1.6506	0.0128
6	Fatigue*	−0.5915	0.2735	−1.1544	−0.0699
7	Sore_throat	−0.2978	0.3701	−1.0415	0.3912
8	Loss_of_smell*	0.8544	0.3889	0.0841	1.6103
9	Runny_nose	0.3771	0.3572	−0.3197	1.0549
10	Cough	−0.1341	0.2910	−0.7123	0.4365
11	Shortness_of_breath	0.0614	0.3703	−0.6873	0.7833
12	Abdominal_pain*	0.4931	0.2230	0.0447	0.9144
13	Respiratory disease	−0.0581	0.4035	−0.8211	0.7439
14	Domestic travel*	0.4235	0.1715	0.1011	0.7661
15	International travel*	1.6074	0.2390	1.1375	2.0692
16	IgG (positive)	0.3766	0.2065	−0.0264	0.7917

*Indicates a 95% credible interval that does not include 0 (i.e., effect credibly different from zero).

**Figure 3:**
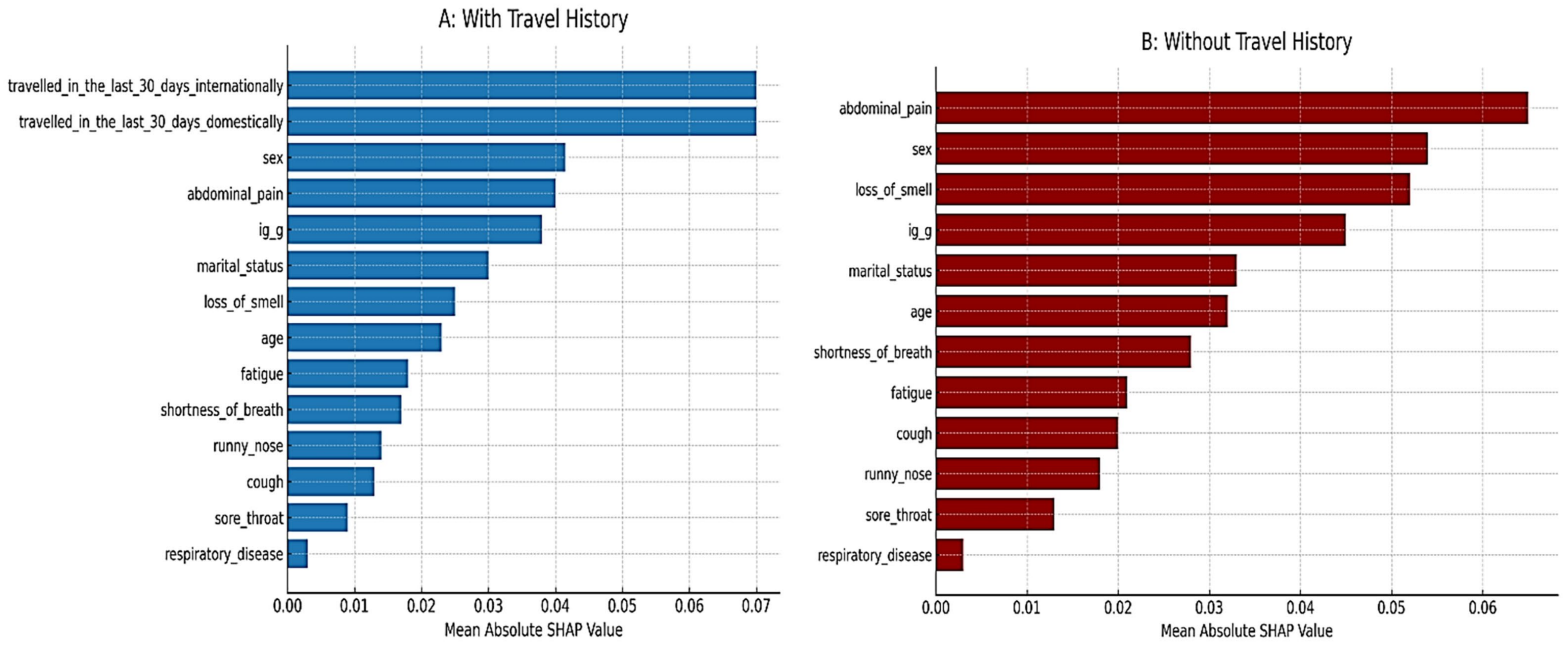
SHAP predictor importance from the random forest models.

**Figure 4:**
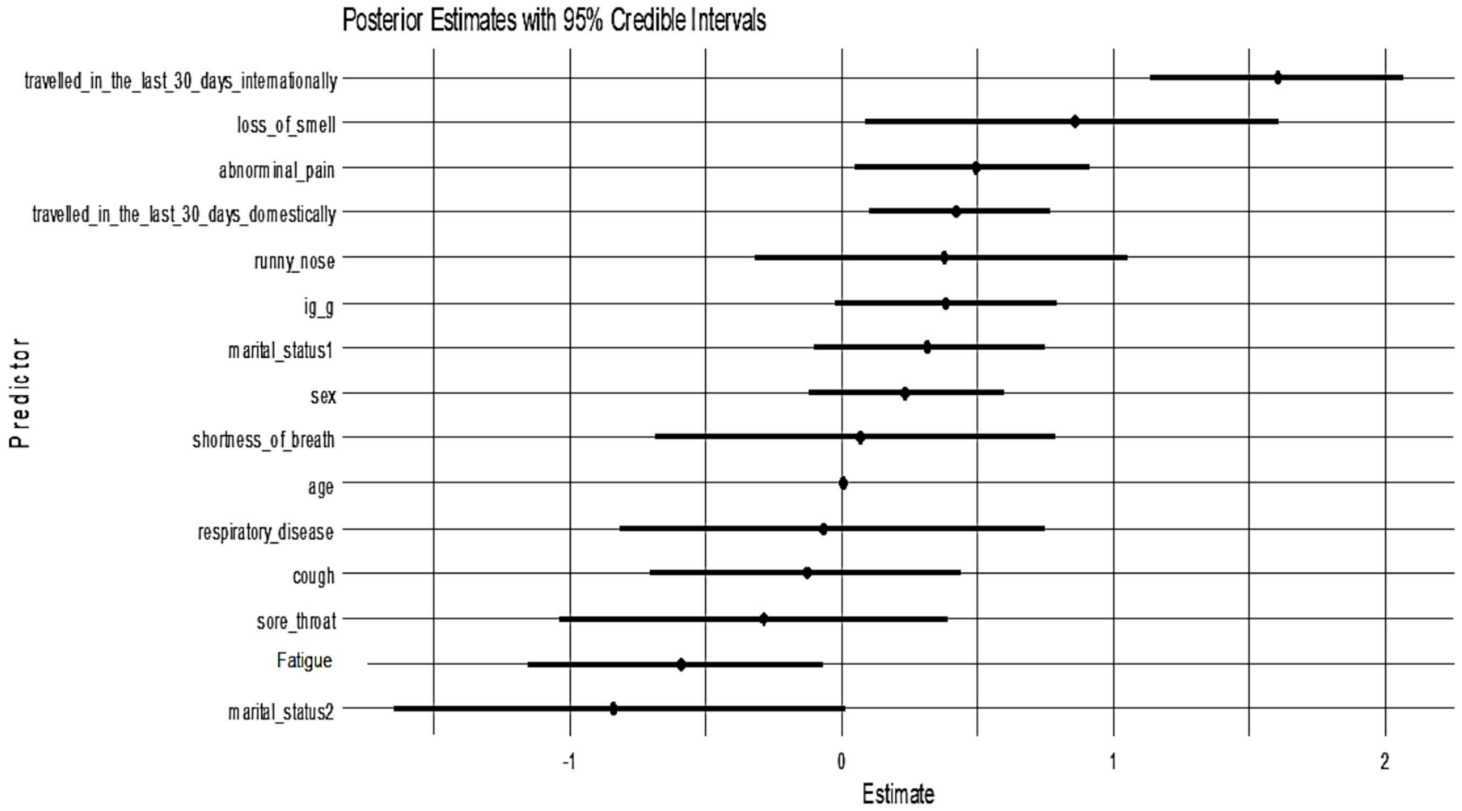
Posterior estimates with 95% credible intervals (Model 1). Predictors with intervals that do not include zero are strongly associated with PCR positivity:

All candidate variables had posterior inclusion probabilities (PIPs) of approximately 1.0 in Model 1, indicating the model consistently included all predictors. Figure 3 shows the posterior coefficient estimates with 95% credible intervals (CrIs) for Model 1. Predictors whose CrIs do not cross zero are considered significant; as shown, international travel, domestic travel, loss of smell, and abdominal pain have CrIs entirely above zero, while age and sex have CrIs spanning zero (Figure 4).

To evaluate the robustness of findings to prior assumptions, a Bayesian logistic regression model was re-estimated using weakly informative Cauchy priors (center = 0, scale = 2.5) following Gelman et al. (2008). This sensitivity model yielded posterior estimates that were largely consistent with the primary model. Notably, international travel within the last 30 days (Estimate = 1.60, 95% CrI: 1.11 to 2.10), presence of loss of smell (Estimate = 0.85, 95% CrI: 0.08 to 1.58), abdominal pain (Estimate = 0.48, 95% CrI: 0.03 to 0.92), and domestic travel (Estimate = 0.42, 95% CrI: 0.09 to 0.77) were associated with increased odds of testing positive for SARS-CoV-2. Conversely, fatigue remained a credible protective factor (Estimate = −0.61, 95% CrI: −1.13 to −0.09). The 95% credible intervals for other predictors overlapped zero, indicating insufficient evidence for strong associations. All parameters demonstrated excellent convergence (Rˆ = 1.00) and high effective sample sizes, confirming model stability and reliability. These results are presented in Table 7 below. All significant predictors in Model 2 were also identified in Model 1, reinforcing the stability of these effects across prior assumptions. Each had a posterior inclusion probability (PIP) of approximately 1.0, confirming their consistent importance across MCMC samples.

**Table 7:** Posterior estimates from Model 2 (Bayesian logistic regression with Cauchy priors).

	Coefficient	Estimate	Est.Error	l-95% CI	u-95% CI	Rhat	Bulk_ESS	Tail_ESS
1	Intercept	−1.2588	0.4521	−2.1393	−0.3848	1.0006	5609.457	3349.905
2	Age	0.0016	0.0067	−0.0115	0.0148	1.0002	5117.734	3790.557
3	Sex (male)	0.2281	0.1890	−0.1356	0.6026	1.0026	7309.019	2884.834
4	Marital_status1 (single)	0.3035	0.2142	−0.1229	0.7228	1.0008	5547.912	3163.030
5	Marital_status2 (widow/widower)*	−0.8920	0.4376	−1.7554	−0.0231	1.0004	6040.356	2866.929
6	Fatigue*	−0.6078	0.2686	−1.1326	−0.0883	1.0011	6213.057	2802.454
7	Sore_throat	−0.2877	0.3833	−1.0503	0.4484	1.0017	5913.666	2851.732
8	Loss_of_smell*	0.8477	0.3823	0.0763	1.5838	1.0012	5669.066	2923.263
9	Runny_nose	0.3675	0.3538	−0.3339	1.0462	0.9998	5226.675	3053.602
10	Cough	−0.1364	0.2901	−0.7087	0.4227	1.0029	6066.075	2985.689
11	Shortness_of_breath	0.0618	0.3647	−0.6581	0.7893	0.9999	5347.596	2839.107
12	Abdominal_pain*	0.4835	0.2225	0.0341	0.9173	1.0004	7313.578	2766.120
13	respiratory_disease	−0.1018	0.4146	−0.8954	0.7330	1.0011	6851.385	2645.507
14	Domestic travel*	0.4224	0.1777	0.0932	0.7731	0.9996	6061.214	2916.948
15	International travel*	1.6003	0.2539	1.1055	2.0975	1.0002	6579.457	2549.944
16	IgG (Positive)	0.3621	0.1947	−0.0134	0.7445	1.0008	8009.948	3141.571

*Indicates a 95% credible interval that does not include 0 (i.e., effect credibly different from zero).

**Figure 5:**
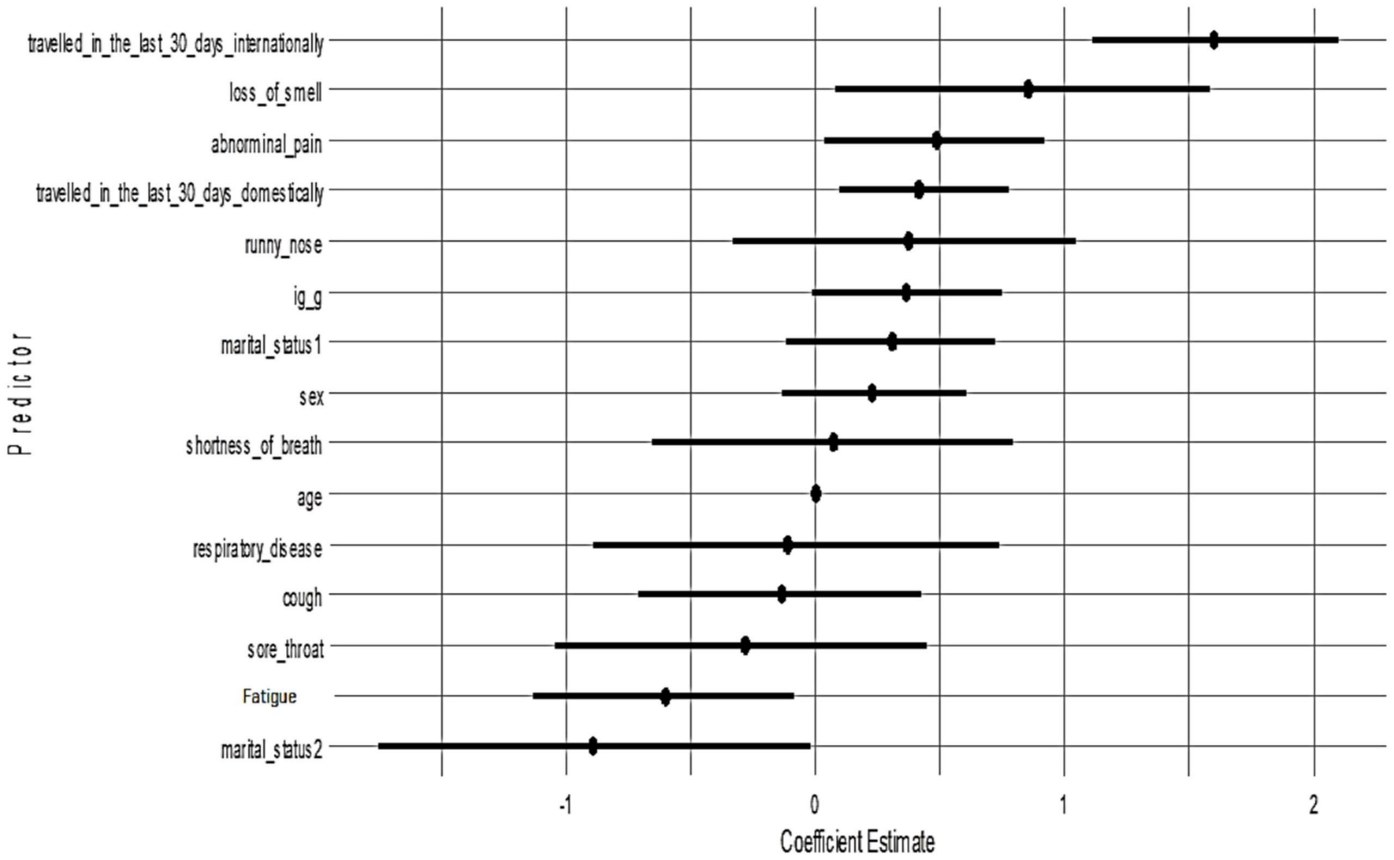
Posterior coefficient estimates with 95% credible intervals (Model 2).

Figure 5 presents the posterior coefficient estimates with 95% credible intervals for Model 2 (Cauchy prior). Loss of smell (posterior median = 0.85, 95% CrI: 0.08–1.58), abdominal pain (0.48, 95% CrI: 0.03–0.92), domestic travel (0.42, 95% CrI: 0.09–0.77), and international travel (1.60, 95% CrI: 1.11–2.10) had credible intervals that excluded zero, indicating strong positive associations with SARS-CoV-2 PCR positivity. Fatigue and marital status (widow/widower) showed significant negative associations. All other predictors had wide credible intervals overlapping zero, suggesting weaker or uncertain effects. The pattern of significant predictors mirrors Model 1, confirming that key associations, particularly travel history and anosmia, are robust to different prior choices.

Table 8 compares the Bayesian logistic regression models using leave-one-out cross-validation (LOO-CV). Model 1 was fitted with moderately informative Normal priors, while Model 2 used weakly informative Cauchy priors. The estimated difference in expected log pointwise predictive density (elpd_diff) between the models was −0.1 with a standard error of 0.2, indicating no meaningful difference in predictive performance. This negligible difference supports that key inferences remain stable even under different prior specifications. Although a few observations had high Pareto *k* values, moment-matched LOO diagnostics confirmed stable performance, supporting the validity of the posterior inferences across both models.

**Table 8:** Model comparison—Bayesian logistic regression.

Model	elpd_diff	se_diff
Model 1 (moderately informative priors)	0.0	0.0
Model 2 (weakly informative Cauchy priors)	−0.1	0.2

To address the class imbalance (74.8% PCR + vs. 25.2% PCR-), SMOTE were applied. Before SMOTE, the dataset contained 711 PCR-positive and 239 PCR-negative cases. After balancing, both classes had 711 observations, ensuring equal representation and reducing bias in model training. Figure 3 shows the SHAP predictor importance rankings from the Random Forest models. Panel A (blue bars) corresponds to the model including travel history, while Panel B (red bars) corresponds to the model excluding travel history. Bars represent mean absolute SHAP values, indicating the relative contribution of each predictor to the prediction of SARS-CoV-2 PCR positivity. In Panel A, recent international and domestic travels were the strongest predictors, followed by sex, abdominal pain, and IgG serostatus. Respiratory disease and sore throat contributed the least. In Panel B, after travel variables were removed, abdominal pain emerged as the most influential predictor, followed by sex, loss of smell, and IgG serostatus. Respiratory disease and sore throat remained low in importance. This pattern highlights how travel history strongly drives model predictions when included, whereas non-travel predictors (e.g., abdominal pain, anosmia, sex) become more dominant when travel data are not available.

Table 1 summarizes the comparative performance of random forest models trained with and without travel history. When travel variables were included, the model achieved 78.6% accuracy and a balanced accuracy of 64.8%. Specificity was high at 92.7%, meaning the model was effective at identifying PCR-negative individuals. However, sensitivity was low at 36.8%, indicating many PCR-positive cases were missed. The F1 score was 0.463, reflecting modest balance between precision and recall under travel-driven predictions. After excluding international and domestic travel predictors, performance improved across several dimensions. Balanced accuracy rose to 81.3%, and sensitivity nearly doubled to 70.8%, suggesting the model became substantially better at identifying true positives. The F1 score increased to 0.790, reflecting stronger alignment between precision and recall. Accuracy also improved modestly to 81.2%, and specificity remained high (91.7%). Both versions of the model showed excellent discrimination: AUC was 0.963 with travel data and 0.947 without. While travel history boosted specificity, removing it produced a more balanced model better suited for flagging infected individuals, especially in settings where travel history is unavailable or unreliable. The shift in performance metrics demonstrates that non-travel predictors, particularly symptoms, IgG serostatus, and demographics; retain strong predictive value.

As summarized in Supplementary Table S3, the Random Forest showed near-zero sensitivity for the minority (negative) class before SMOTE (reported as specificity = 0.248–0.262) with low balanced accuracy (0.569–0.571), despite a high AUC (0.943–0.948). After SMOTE, negative-class sensitivity (specificity) rose to 0.904–0.917 and balanced accuracy to 0.800–0.813, while AUC remained essentially unchanged (0.939–0.947). Logistic and Bayesian models were not SMOTE-adjusted and showed stable metrics across imputed vs. complete-case analyses (Tables 5, 7; Supplementary Table S3).

Figure 1 compares the diagnostic performance of three predictive models using ROC curves. The frequentist logistic regression model had an AUC of 0.73, indicating moderate ability to distinguish PCR-positive from PCR-negative cases. In contrast, the random forest model including travel history performed substantially better, achieving an AUC of 0.96 and demonstrating excellent discriminative power. After removing international and domestic travel variables, the model still maintained a strong AUC of 0.95, confirming that symptoms, demographic factors, and IgG serostatus alone are sufficient for high classification accuracy. Although travel history improved specificity slightly (92.7% vs. 91.7%), it was associated with lower sensitivity (36.8%). Excluding travel predictors nearly doubled sensitivity to 70.8% and increased balanced accuracy to 81.3%, while maintaining high specificity and AUC. This supports the generalizability of a non-travel-based model when travel data are limited or unavailable.

## 4 Discussion

The results of this comparative analysis of frequentist, Bayesian, and ML methods for predicting SARS-CoV-2 PCR positivity provide valuable insights into the effectiveness of each approach. All models identified recent travel history, especially international travel, and the loss of smell (anosmia) as the strongest predictors of a positive PCR test. In the frequentist logistic regression, recent international travel was associated with the highest odds of testing positive (OR = 4.8), followed by domestic travel (OR = 1.5), while self-reported loss of smell also markedly increased the odds (OR = 2.3). These findings align with prior knowledge: travel history was a well-recognized risk factor early in the pandemic, with one study reporting an approximately fourfold increase in odds of COVID-19 infection among individuals with recent travel (Menni et al., 2020; Gu et al., 2020; Kang et al., 2022). In fact, Gu et al. (2020) observed that recent travel; particularly to COVID-19 hotspots, was the most common association with new positive cases, leading the authors to recommend prioritizing travelers for testing when resources are limited. Likewise, sudden loss of smell has been widely reported as a distinctive symptom of COVID-19. Gerkin et al. (2020) found that acute olfactory loss was the single best predictor of COVID-19 infection among people with respiratory symptoms (ROC AUC = 0.72). They even proposed a simple olfactory rating scale as a screening tool when PCR tests are impractical (Gerkin et al., 2020). Our results corroborate these patterns: recent smell loss and travel exposure are critical red flags, reinforcing their usefulness in triaging patients when immediate PCR testing is unavailable.

Beyond these key predictors, our models provided additional insights. The frequentist logistic model indicated that abdominal pain, a somewhat atypical symptom, was significantly associated with PCR positivity (OR = 1.6), whereas a very common symptom like fatigue showed a negative association with positivity (OR = 0.54). At first glance, a negative coefficient for fatigue seems counterintuitive, since fatigue is common in COVID-19. A possible explanation is multicollinearity and symptom clustering: fatigue was almost ubiquitous in our cohort (84.4% prevalence) and is a non-specific complaint; individuals who did not report fatigue may have had other more specific symptoms (like anosmia) driving their COVID-19 diagnoses. In a multivariable model, this can make fatigue appear protective; essentially highlighting that lack of fatigue (in the presence of other symptoms) might distinguish some COVID-19 cases. This phenomenon has precedent in other analyses. For example, an analysis of over 67,000 cases in Argentina found that classical respiratory complaints (e.g., dyspnea, chest pain, even abdominal pain) were negatively or non-significantly associated with COVID-19 positivity in multivariate models (Fleitas et al., 2021; Park, 2021). Instead, anosmia and dysgeusia (loss of taste) were among the strongest positive predictors, consistent across age groups (Fleitas et al., 2021; Mutiawati et al., 2021; Carignan et al., 2020). Thus, our finding that a very common symptom (fatigue) did not increase predictive power, and even showed a negative coefficient in a multivariate context, aligns with the idea that specific symptoms (anosmia, etc.) carry more diagnostic weight than broadly prevalent ones (Menni et al., 2020). The association of abdominal pain with higher odds of COVID-19 in our study is intriguing. Gastrointestinal manifestations of COVID-19 are well documented but generally less frequent than respiratory symptoms (Patel et al., 2022; Schmulson et al., 2020). Typical studies report only about 4%–7% of COVID-19 patients experience abdominal pain (Perisetti et al., 2020). In our cohort, however, a striking 72.6% reported abdominal pain, which is an unusually high prevalence. This discrepancy could be due to differences in data collection (active symptom querying in our study leading to more reports of mild pain) or possibly a unique predictor of our sample or setting. Some GI-focused analyses noted that as the pandemic progressed, clinicians became more aware of gastrointestinal symptoms and thus reported them more frequently (Perisetti et al., 2020; Akin et al., 2020). It’s also possible that, in a cohort enriched with travelers or specific exposures, GI symptoms were particularly common. Regardless, our finding suggests that when abdominal discomfort is widely present in a group under investigation, it may help flag COVID-19 cases, a point that contrasts with at least one study where abdominal pain was not a useful discriminator (Fleitas et al., 2021). We acknowledge that the high rate of reported abdominal pain in our data could reflect reporting or interpretation bias. Participants or clinicians might have interpreted general malaise as “abdominal pain,” or there may have been overlap with other gastrointestinal issues not unique to COVID-19. It is also important to note that many common COVID-19 symptoms (e.g., fatigue, cough) are non-specific and can be caused by other illnesses such as influenza. Therefore, while symptom-based screening is valuable for detecting likely positives, it may also capture cases of other respiratory infections, limiting its specificity when those alternative diagnoses are present (Perisetti et al., 2020).

Demographic factors played a smaller role in our models. Neither age nor sex was significantly associated with PCR positivity in the frequentist or Bayesian regressions (Tables 4, 6, 7), which is consistent with some epidemiological data indicating that, while older age and male sex are risk factors for severe COVID-19 outcomes, they do not always strongly differentiate infection probability in a young, predominantly healthy screening population (Molani et al., 2022). Interestingly, the classical frequentist model did find that being widowed was associated with lower odds of testing positive. We interpret this cautiously: widowed individuals made up only 3% of our sample, and they tend to be older; this result may reflect lower exposure risk or mobility in that subgroup, or simply be a spurious finding. We note it here for completeness, but it is likely not a generalizable predictor of COVID-19 risk. In contrast, the machine learning model ranked sex as a relatively influential predictor (with female sex associated with higher SHAP importance for positivity). This discrepancy between models suggests that the random forest may be capturing subtle nonlinear interactions involving sex; for instance, perhaps female participants in our sample had different symptom patterns or exposure contexts, even though sex by itself did not show a main effect in logistic regression. Prior studies have reported mixed results on sex differences in COVID-19 infection rates; some analyses early in the pandemic found males and females to be infected at similar rates even if males had worse clinical outcomes, while others noted context-dependent differences (Fleitas et al., 2021; Doerre & Doblhammer, 2022; Sieurin et al., 2022). Our findings do not provide strong evidence of a sex-based infection risk disparity, but the machine learning model’s inclusion of sex in its top predictors suggests there may be interaction effects worth further exploration (for example, certain symptoms might have different predictive value in men vs. women, a nuance that a tree-based model could capture) (Zoabi et al., 2021; Azizi et al., 2022).

One of the aims of this study was to compare how a traditional frequentist regression, a Bayesian regression, and a machine learning model perform on the same prediction task. Overall, we found that all three approaches identified a consistent set of important predictors (travel history, anosmia, etc.), which speaks to the robustness of these predictors. The Bayesian approach, by incorporating prior information, did not drastically change the point estimates obtained by the frequentist model rather, it shrank some coefficients slightly towards zero and produced 95% credible intervals that explicitly reflect uncertainty. For key predictors like international travel or loss of smell, the Bayesian posterior remained far from zero despite the prior, indicating strong data-driven effects. For others (like age, sex) that had weak effects, the Bayesian credible intervals comfortably included zero, highlighting our uncertainty about those associations. The Bayesian models also allowed us to quantify the posterior inclusion probability (PIP) of each predictor. In our analysis, all chosen predictors had PIP = 1.0, meaning they were consistently retained across MCMC samples and contributed to the posterior estimates. This was expected given our deliberate variable selection, but it is reassuring that there were no “junk” variables with PIP < 1. Importantly, we found that changing the prior, from a moderately informative Normal prior to a weakly informative Cauchy prior, did not materially alter the results (Table 8). This sensitivity analysis increases our confidence that the conclusions are not an artifact of a particular prior assumption. One benefit of the Bayesian framework is the probabilistic interpretation of results: for example, we can say there is roughly a 95% probability that the effect of recent international travel on the log-odds of COVID-19 positivity lies between about 1.1 and 2.1 (which corresponds to roughly a 3- to 8-fold increase in odds) given our data and prior assumptions. Such an interpretation may be more intuitively appealing to clinicians than a frequentist confidence interval, which does not have a direct probability meaning. Additionally, the Bayesian approach highlights the capability to incorporate prior knowledge (had we possessed strong prior beliefs about certain predictors) which can be valuable in scenarios of sparse data or expert-driven hypotheses (Sullivan et al., 2025).

In terms of predictive performance, the machine learning model (random forest) notably achieved the highest discriminative ability (cross-validated AUC = 0.947–0.963), outperforming both logistic regression approaches. This level of performance indicates excellent classification of PCR outcomes (Couronné et al., 2018; Sundaravadivel et al., 2025; Leonard et al., 2022). In fact, our random forest’s AUC is on par with or better than the best results reported in the literature for symptom-based COVID-19 prediction models (Zoabi et al., 2021; Pal et al., 2022; Rashidi et al., 2024; Galoustian, 2022). For instance, one automated machine learning approach that combined clinical and laboratory predictors achieved 95.6% sensitivity and 98% specificity in classifying COVID-19 status (Rashidi et al., 2024). Another study that used simple symptom and demographic predictors across five different ML algorithms reported AUC values above 0.81, with their best models reaching 76%–81% accuracy (Galoustian, 2022). Similarly, Lanzilao et al. (2023) observed that machine learning methods, particularly random forests and logistic regression, achieved high discrimination (AUC > 0.80) in COVID-19 prediction tasks using routine clinical and laboratory data, closely aligning with our performance metrics (Lanzilao et al., 2023; Daghistani & Alshammari, 2020). Our random forest model slightly exceeds these benchmarks in AUC, likely reflecting the richness of our predictor set (including travel history and detailed symptomatology) and the power of ensemble methods in capturing complex patterns. Notably, the random forest can implicitly model interactions and non-linear relationships that a single logistic regression might miss (for example, interactions between specific symptoms and exposures). Indeed, we suspect that the RF learned a rule-like pattern: “IF recent international travel = yes, then high likelihood of COVID.” Consistent with that, SHAP predictor importance analysis showed that international and domestic travel were the top two predictors when they were included, indicating the model heavily used those predictors for its decisions. Before balancing, the model exhibited near-zero sensitivity for PCR-negative cases due to the 3:1 class imbalance; this was corrected after SMOTE (Table 1; Supplementary Table S3). However, this strength also proved to be a double-edged sword. The initial RF model, while overall highly accurate, exhibited an imbalance in its error pattern, it showed very high specificity (>92%) but relatively low sensitivity (37%). In other words, it was very good at flagging negatives (especially those with no travel history and mild symptoms) but missed a substantial portion of positives. Upon investigation, we discovered that the model was over-relying on travel history: many PCR-positive individuals in our dataset had traveled, so the RF effectively learned to associate “no travel” with being negative. As a result, positive cases without a travel history were often misclassified as negative. We tested this by removing the travel-related predictors and retraining the model. The outcome was striking: the model’s sensitivity improved dramatically (from 37% to 71%), while maintaining a high specificity (92%), and the balanced accuracy jumped from 64.8% to 81.3%. Initially, this might seem counter-intuitive one would expect removing an important predictor to degrade performance, but in this case the change forced the model to base predictions on symptoms and demographics alone, making it more generalizable. The “without travel” model did have a slightly lower specificity (it produced a few more false positives among those with no travel history and mild symptoms), but it missed far fewer positives (i.e., it greatly reduced the false negatives) compared to the original model. In practical terms, this means that symptoms and basic demographics carried substantial predictive signal on their own, and the model became much better at catching positive cases across the board once it wasn’t single-mindedly focused on travel. From an implementation standpoint, this has important implications. In scenarios where travel history is not readily available or not relevant (e.g., later in the pandemic when community spread dominates), a symptom-only model might actually perform better in identifying cases, as our analysis suggests. Including travel data can boost precision (improving specificity by reducing false alarms, since travel is a strong risk factor when it applies) but at the cost of missing positives who do not fit that profile. This highlights the importance of context in deploying prediction models, our full model (with travel variables) would be ideal in an early containment phase to flag high-risk travelers, whereas the symptom-only model might generalize better in a widespread community transmission phase or when reliable travel/exposure data are unavailable. Another consideration is that removing travel history only slightly reduced the overall AUC of the random forest (from 0.963 to 0.947, a minor drop), demonstrating strong performance even without travel data. Indeed, in the travel-excluded model, predictors like abdominal pain, loss of smell, sex, and IgG serostatus became the primary drivers (as illustrated in Figure 1), and achieving 0.947 AUC with those predictors alone is encouraging, it implies that even without knowing travel history, a data-driven model can perform very well. Including travel gave a tiny edge in AUC, but as discussed, it came with the trade-off of reduced sensitivity in our context.

Overall, the random forest’s superior performance is not surprising given its flexibility and the potential interactions in the data (for instance, combinations of symptoms that jointly predict infection beyond their individual effects). Machine learning models are often criticized as “black boxes,” but we mitigated this by using SHAP values to interpret predictor importance. After removing travel, the most influential predictors in the random forest were abdominal pain, sex, loss of smell, and IgG serostatus, which is consistent with the regression findings (except that sex appears more important in the RF, possibly due to the interactions as noted). SHAP analysis also confirmed that variables like respiratory disease history and sore throat contributed the least to the model, consistent with their minimal effects in the logistic regression. This interpretability step lends transparency to the model: for example, we can explain an individual prediction by noting whether the presence of certain symptoms (e.g., anosmia, GI complaints) or patient factors (e.g., female sex, lack of prior IgG antibodies) pushed the probability of positivity higher. Ultimately, our random forest model, especially in its travel-excluded form, demonstrates that machine learning can produce a highly accurate COVID-19 screening tool using readily available clinical information (Sievering et al., 2022). The logistic regression models, in turn, provide assurance that the relationships identified are medically sensible and not artifacts of overfitting, since both frequentist and Bayesian approaches converged on the same key predictors and effect directions.

Our findings have practical implications for how predictive models might be used in a pandemic context. In outbreaks or low-resource settings, a predictive algorithm can flag likely COVID-19 cases for priority PCR testing or immediate isolation. PCR remains the diagnostic gold standard for COVID-19 (Parker & Boyer, 2022), but it requires laboratory processing (often causing days-long delays) and large-scale PCR testing can be impractical in surges (Lundon et al., 2021). For example, one risk stratification model (trained on data from the Mount Sinai Health System) achieved a negative predictive value of 96% at a high-risk cutoff, providing a “superior net benefit” over blanket testing and thus “conserving vital resources” (Lundon et al., 2021). Similar triage scores (e.g., for healthcare workers) have been developed to guide testing under resource constraints (Hohl et al., 2022). In practice, the performance of our model, sensitivity 71% and specificity 92% in the travel-excluded random forest, means far fewer cases are missed compared to using symptoms alone without a model, while many low-risk individuals can safely defer or avoid testing (allowing limited PCR tests to be focused on the high-probability group). Including recent travel or known exposure history can of course boost screening yield in early epidemic phases. In one San Francisco study, 43% of early COVID-19 cases were travel-related (Gu et al., 2020), so testing efforts initially focused on travelers or close contacts. Our findings reflect this pattern: a travel variable was highly predictive in our data collected during a containment phase. However, we also show that symptom-based models remain robust even if travel/exposure data are later unavailable. In other words, exposure history can improve case finding when available (Gu et al., 2020), but the core symptom algorithm still works well without it. We also found that including anti-SARS-CoV-2 IgG serostatus in the model is technically feasible but of secondary importance. This aligns with public health guidelines noting that high population seroprevalence (past infection rates) limits antibody testing’s acute diagnostic value (Hayden et al., 2024). By the time of our study, a positive IgG was more likely to reflect prior infection or immunity rather than an active infection (Hayden et al., 2024). Notably, higher antibody titers do correlate with lower infection risk (Hayden et al., 2024), so knowing someone’s IgG level might slightly adjust their prior probability of acute COVID-19. In future scenarios (e.g., highly vaccinated or previously exposed populations), serostatus could help refine screening probabilities, but it cannot replace molecular testing for diagnosing current infection.

We verified our predictors using both frequentist and Bayesian methods, and they agree, increasing confidence that the signals we identified (e.g., anosmia, travel, gastrointestinal symptoms) are real and not modeling artifacts. The superior accuracy of our machine learning models suggests that health systems might consider deploying similar algorithms as part of screening and triage protocols. Of course, caution is warranted: models must be continuously re-evaluated as the virus evolves. Symptom importance can shift over time, for example, loss of smell was very common in early 2020 strains but became much rarer with the Omicron variant (only 13% of Omicron cases had anosmia versus 34% of cases with the earlier Delta variant) (Rodriguez-Sevilla et al., 2022). Any deployed screening tool will therefore require ongoing monitoring, validation on new patient data, and periodic retraining to maintain its effectiveness in the face of such changes.

## 5 Conclusion

In our analysis of SARS-CoV-2 screening data, frequentist and Bayesian logistic regression and a machine learning (random forest) model all identified the same key predictors of PCR positivity, recent travel history and loss of smell were the strongest signals, along with symptoms like abdominal pain. The logistic regression models provided clear, interpretable risk factors that align with known COVID-19 predictors, and the Bayesian analysis confirmed these results with probabilistic uncertainty estimates. The random forest model achieved the highest accuracy (cross-validated AUC = 0.963), demonstrating that machine learning can leverage non-linear patterns and interactions to improve prediction. These findings suggest that a symptom-based triage tool could effectively flag high-risk individuals for confirmatory PCR testing. Importantly, such a tool would complement, not replace PCR diagnostics. Flagged individuals should still receive PCR confirmation, but the model’s predictions can help prioritize limited testing resources by identifying likely positives. In practice, a hybrid approach may be ideal, for example, a quick scoring system derived from the logistic regression for use in the field, backed by a more complex ML algorithm for fine-tuning decisions in borderline cases (with SHAP or similar methods providing explanation). Overall, our results show that data-driven models can augment COVID-19 testing strategies by rapidly identifying likely cases and informing resource allocation.

### 5.1 Study limitations

We acknowledge several limitations in our study. First, our analysis is based on a dataset drawn from a high-prevalence screening context (75% PCR-positive) with a large proportion of recent travelers, which may limit the generalizability of our findings to broader community settings. In a more typical population with lower infection prevalence and different exposure patterns, the model’s positive predictive value would likely be lower, and its calibration might need adjustment. Second, our predictive predictors were restricted to self-reported symptoms, basic demographics, travel history, and a single immunological marker (IgG status). We did not incorporate potentially important data such as vital signs, detailed medical histories, comorbidities, or known exposure events, which could improve model accuracy and robustness if included. Third, self-report and recall bias may affect symptom data quality: participants or clinicians reported symptoms subjectively, which can introduce noise or inconsistencies. For example, fatigue and abdominal pain were reported at very high rates in our sample (as discussed), suggesting possible over-reporting, interpretation differences, or selection bias in who was tested; moreover, these symptoms could have been due to other circulating illnesses (e.g., influenza), potentially leading to false positives in contexts where COVID-19 is not the only prevalent infection. Fourth, the retrospective cross-sectional design and our use of cross-validation on the same dataset present a risk of overfitting to idiosyncrasies in our data, the model might have inadvertently learned predictors (such as the travel history effect) too specifically, limiting its performance on new data. Additionally, the pandemic context was evolving during our study period, and factors like new viral variants or increasing vaccination rates could alter the symptom profile or prevalence of disease over time. This means the model would require ongoing recalibration to remain accurate. Finally, we urge caution in interpreting the observed associations as causal. For instance, a negative coefficient for fatigue or a positive coefficient for being widowed in the regression models may reflect subgroup effects or collinearity rather than true protective or risk factors. Our models identify predictive associations, but these should not be misconstrued as evidence that certain factors *cause* or *prevent* infection; rather, they aid in prediction given the data context.

### 5.2 Future work

Looking ahead, future work will focus on enhancing the model’s generalizability, robustness, and practical utility. First, we plan to perform external validation by testing and recalibrating our models on new datasets from different populations and geographic regions, including data from post-vaccination eras and involving new viral variants, to ensure that our findings and model performance remain valid under diverse conditions. Second, we will incorporate additional predictive predictors such as vital signs, laboratory or rapid test results, comorbidity information, and documented exposure history, which could further improve predictive power and reliability. Third, we aim to explore methods for real-time model updating using continuously streaming data, so that the model can adapt as local epidemiological conditions change (for example, adjusting to shifts in prevalent strains or population immunity). Finally, we will prioritize usability and deployment considerations by developing a user-friendly interface (such as a smartphone app or web-based calculator) and integrating the tool into clinical and public health workflows. These efforts will help make our predictive screening tool more accurate, practical, and impactful for real-world use.

## Data Availability

The original contributions presented in the study are included in the article/Supplementary material, further inquiries can be directed to the corresponding author.
