# Supplementary material for: Comparative analysis of frequentist, Bayesian, and machine learning models for predicting SARS-CoV-2 PCR positivity
Source: Front Artif Intell. 2025 Dec 3;8:1668477. doi: 10.3389/frai.2025.1668477 (PMC12708910; doi:10.3389/frai.2025.1668477)
Supplement: Supplementary file 1 [file Data_Sheet_1.PDF]

Table S2. Variable Missingness Summary

Variable	Type	Missing Count	Missing (%)	Imputation Method
Age	Continuous	18	1.9	Median
Sex	Categorical	10	1.1	Mode
Marital Status	Categorical	12	1.3	Mode
Fatigue	Categorical	14	1.5	Mode
Loss of Smell	Categorical	9	0.9	Mode
Runny Nose	Categorical	11	1.2	Mode
Shortness of Breath	Categorical	7	0.7	Mode
Abdominal Pain	Categorical	16	1.7	Mode
Cough	Categorical	13	1.4	Mode
Sore Throat	Categorical	8	0.8	Mode
Domestic Travel	Categorical	15	1.6	Mode
International Travel	Categorical	10	1.1	Mode
IgG Serostatus	Categorical	6	0.6	Mode
Respiratory Disease History	Categorical	5	0.5	Mode

Table S3: Sensitivity analysis of model performance showing effects of imputation and SMOTE.

Model	Setting	Metric	Imputed	Complete-case	Absolute Δ
Frequentist Logistic Regression	Original class distribution	AUC	0.728	0.721	0.007
		Accuracy	0.797	0.784	0.013
		Sensitivity (Positive)	0.961	0.952	0.009
		Specificity (Negative)	0.310	0.326	0.016
		Balanced Accuracy	0.636	0.639	0.003
		F1	0.805	0.796	0.009
Bayesian Logistic Regression	Original class distribution	AUC	0.731	0.724	0.007
		Accuracy	0.795	0.781	0.014
		Sensitivity (Positive)	0.958	0.949	0.009
		Specificity (Negative)	0.318	0.333	0.015
		Balanced Accuracy	0.638	0.641	0.003
		F1	0.803	0.792	0.011
Random Forest	Pre-SMOTE	AUC	0.948	0.943	0.005
		Accuracy	0.776	0.764	0.012
		Sensitivity (Positive)	0.889	0.879	0.010
		Specificity (Negative)	0.248	0.262	0.014
		Balanced Accuracy	0.569	0.571	0.002
		F1	0.822	0.812	0.010
Random Forest	Post-SMOTE	AUC	0.947	0.939	0.008
		Accuracy	0.812	0.801	0.011
		Sensitivity (Positive)	0.708	0.696	0.012
		Specificity (Negative)	0.917	0.904	0.013
		Balanced Accuracy	0.813	0.800	0.013
		F1	0.790	0.778	0.012

Frequentist and Bayesian logistic regression evaluated under the original class distribution (SMOTE not applied to regression models).
Random Forest evaluated before and after SMOTE, each reported for imputed and complete-case datasets. SMOTE markedly improves minority-class (PCR-negative) sensitivity and balanced accuracy while preserving AUC.
Sensitivity (Positive) = recall for PCR-positive; Specificity (Negative) = recall for PCR-negative; Absolute Δ = {Imputed - Complete-case}.
